# Cytotoxic Activity and Apoptosis-Inducing Potential of Di-spiropyrrolidino and Di-spiropyrrolizidino Oxindole Andrographolide Derivatives

**DOI:** 10.1371/journal.pone.0058055

**Published:** 2013-03-05

**Authors:** Sumit Kumar Dey, Dipayan Bose, Abhijit Hazra, Subhendu Naskar, Abhishek Nandy, Rudra Narayan Munda, Subhadip Das, Nabanita Chatterjee, Nirup Bikash Mondal, Sukdeb Banerjee, Krishna Das Saha

**Affiliations:** 1 Cancer and Cell Biology Division, CSIR-Indian Institute of Chemical Biology, Council of Scientific and Industrial Research, Kolkata, India; 2 Chemistry Division, CSIR-Indian Institute of Chemical Biology, Council of Scientific and Industrial Research, Kolkata, India; Bauer Research Foundation, United States of America

## Abstract

Anticancer role of andrographolide is well documented. To find novel potent derivatives with improved cytotoxicity than andrographolide on cancer cells, two series of di-spiropyrrolidino- and di-spiropyrrolizidino oxindole andrographolide derivatives prepared by cyclo-addition of azomethine ylide along with sarcosine or proline (viz. sarcosine and proline series respectively) and substitution of different functional groups (-CH3, -OCH3 and halogens) were examined for their cytotoxic effect on a panel of six human cancer cell lines (colorectal carcinoma HCT116 cells, pancreatic carcinoma MiaPaCa-2 cells, hepatocarcinoma HepG2 cells, cervical carcinoma HeLa cells, lung carcinoma A549 and melanoma A375 cells). Except halogen substituted derivatives of proline series (viz. CY2, CY14 and CY15 for Br, Cl and I substitution respectively), none of the other derivatives showed improved cytotoxicity than andrographolide in the cancer cell lines examined. Order of cytotoxicity of the potent compounds is CY2>CY14>CY15>andrographolide. Higher toxicity was observed in HCT116, MiaPaCa-2 and HepG2 cells. CY2, induced death of HCT116 (GI_50_ 10.5), MiaPaCa-2 (GI_50_ 11.2) and HepG2 (GI_50_ 16.6) cells were associated with cell rounding, nuclear fragmentation and increased percentage of apoptotic cells, cell cycle arrest at G1 phase, ROS generation, and involvement of mitochondrial pathway. Upregulation of Bax, Bad, p53, caspases-3,-9 and cleaved PARP; downregulation of Bcl-2, cytosolic NF-κB p65, PI3K and p-Akt; translocation of P53/P21, NF-κB p65 were seen in CY2 treated HCT116 cells. Thus, three halogenated di-spiropyrrolizidino oxindole derivatives of andrographolide are found to be more cytotoxic than andrographolide in some cancer cells. The most potent derivative, CY2 induced death of the cancer cells involves ROS dependent mitochondrial pathway like andrographolide.

## Introduction

Andrographolide, a diterpenoid lactone isolated from *Andrographis paniculata,* known as ‘the King of Bitters’, exhibits several pharmacological activities including immuno-stimulation, cytotoxicity, anti-inflammation, anticancer effect, hypotensive action cardio-protective action HIV [1]–[11]. Though, reports on anticancer role of andrographolide are rapidly increasing, there are limited reports with its derivatives. Jada *et al*. have reported antitumor activities of benzylidene derivatives of andrographolide in breast and colon cancer models [12]. Pyrrolidine-2-spiro-3′-oxindole ring system is an important building block of different natural products as well as pharmaceuticals. Hazra *et al.* have reported the synthesis of different novel di-spiropyrrolidino and di-spiropyrrolizidino oxindole andrographolide analogues (named as sarcosine and proline series respectively) [13]. In the present study, we studied the anticancer role of these di-spyropyrrolidino oxindole and di- spyropyrrolizidino oxindole analogues of andrographolide. As apoptosis is the physiologically desired pathway of cell death by the anticancer agents [14], [15], we wanted to explore the involvements of apoptosis in the andrographolide derivatives induced cell death.

Apoptosis or programmed cell death is a specific form of cell death which plays a crucial role to maintain the integrity of multi cellular organisms. Alterations in the apoptotic pathways are intimately involved in the development of cancer. Cancer is a leading cause of death worldwide [16]. Induction of apoptosis in the hyper proliferating cancer cells by compounds derived from biological sources which are expected to have minimum or no cytotoxic effects on peripheral blood mononuclear cells (PBMC) is the main focus of cancer treatment today (Fig. S6) [17], [18].

Apoptosis also plays a role in preventing cancer; if a cell is unable to undergo apoptosis, due to mutation or biochemical inhibition, it can continue dividing and develop into a tumor. Therefore apoptosis is required by living organisms to conserve homeostasis as well as to maintain their internal states within certain limits.

Apoptosis is characterized by a number of distinct cellular changes such as cell shrinkage, irregularities in cell shape, membrane blebbing, externalization of phosphatidyl serine in cell membrane, chromatin condensation, and inter-nucleosomal DNA fragmentation and increased mitochondrial membrane permeability leading to the release of proapoptotic proteins (like Bad, Bax and caspases) in the cytoplasm and subsequent formation of “apoptotic bodies” (several membrane-enclosed vesicles containing intracellular materials inside). In fact the apoptotic process is functionally conserved and physiological forms of this type of cell death are genetically programmed [19], [20].

Reactive oxygen species (ROS) is an important mediator of DNA damage. DNA damage activates P53, a transcription factor which is transported to the nucleus and transcribes many genes that are necessary for apoptosis induction [21].

The intrinsic or the mitochondrial death pathway is dominated by a cascade of pro- and antiapoptotic Bcl-2 family member proteins [22]–[24]. Pro-apoptotic Bax protein in its activated form undergoes a conformational change resulting in pores in the mitochondrial membrane [25]. This leads to loss of mitochondrial membrane potential and release of cytochrome c in the cytosol and activation of pro-apoptotic caspases [26]–[30]. Once cleaved, the DNA repairing enzyme PARP (poly-ADP-ribose polymerase), no longer supports DNA repairing, resulting in fragmentation of DNA [31]–[34]. Tumor suppressor protein P53 in its activated form regulates many target genes [35], [36]. Translocation of NF-κB subunits such as p65, p50 and c-Rel to the nucleus promotes survival of the cell. Whereas, inhibition of nuclear translocation of NF-κB sub-units, promotes apoptosis. Upregulation of p53 and downregulation of PI3K, p-Akt, NF-κB p65 and MMP-9 proteins are generally associated with apoptosis. It is known that P53 contributes to the decision-making growth arrest and apoptosis. This tumor suppressor protein is known to mediate growth arrest involving P21 as a major effecter [37]. The protein P21 has been shown to induce tumor cell growth arrest and apoptosis [38], [39]. In fact, P53-dependent induction of P21 prevents the entry of cells into S phase [40]. Also inhibition of MMP-9 and MMP-2 inhibits angiogenesis in tumor growth. These biochemical changes are observed in most of the drug-induced apoptosis cases. Therapy targeting these molecules has become a novel strategy for cancer treatment.

## Results

### Chemistry

**Figure 1:**
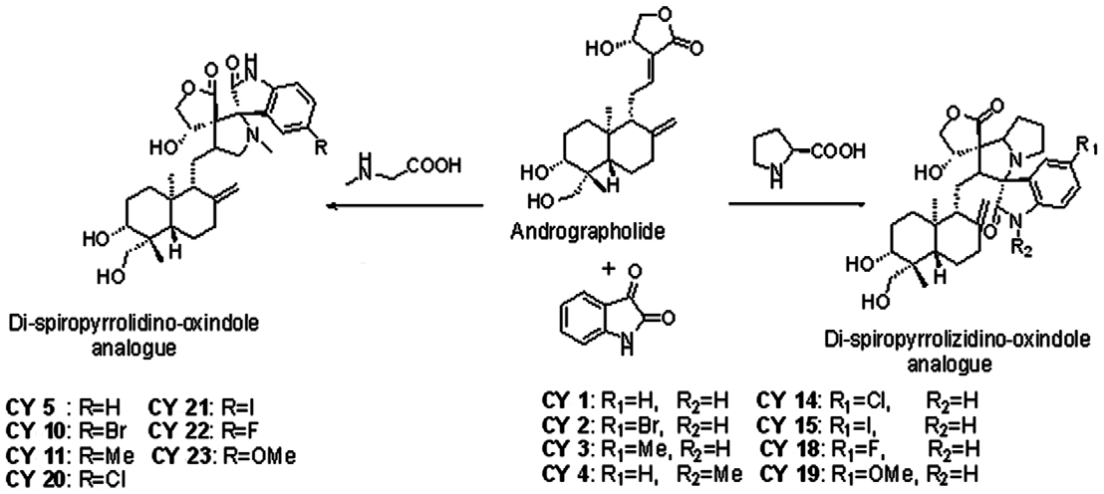
Structure of andrographolide and its derivatives.

Different di-spiropyrrolidino and di-spiropyrrolizidino oxindole andrographolide analogues were prepared as described previously [13]. Seven adducts formed by cycloaddition of azomethine ylide derived from isatin and sarcosine (called here sarcosine series) at 12,13 double bond of andrographolide were named as CY5, CY10, CY11, CY20, CY21, CY22, CY23 (Fig. 1). Eight adducts formed by cycloaddition of azomethine ylide from isatin and proline at 12,13 double bond of andrographolide (called here proline series) were named as CY1, CY2, CY3, CY4, CY14, CY15, CY18 and CY19 (Fig. 1). High Resolution Mass Spectroscopy (HRMS, ESI-MS, positive mode) of the andrographolide derivatives indicating the molecular weight and formula for each analog were shown in Table 1.

**Table 1:** High Resolution Mass Spectroscopic (HRMS) data of different andrographolide derivatives.

HRMS [ESI-MS, positive mode]		
derivatives	m/z	MF
***sarcosin series***		
**CY5**	525.2943 [M+H]^+^[calcd. 525.2965]	C_30_H_40_N_2_O_6_
**CY10**	625.1905 [M+Na]^+^[calcd. 625.1889]	C_30_H_39_BrN_2_O_6_
**CY11**	539.3111 [M+H]^+^[calcd. 539.3121]	C_31_H_42_N_2_O_6_
**CY20**	559.2580 [M+H]^+^[calcd. 559.2575]	C_30_H_39_ClN_2_O_6_
**CY21**	651.1926 [M+H]^+^[calcd. 651.1931]	C_30_H_39_IN_2_O_6_
**CY22**	543.2888 [M+H]^+^[calcd. 543.2870]	C_30_H_39_FN_2_O_6_
**CY23**	577.2880 [M+Na]^+^[calcd. 577.2890]	C_31_H_42_N_2_O_7_
***proline series***		
**CY1**	573.2940 [M+Na]^+^[calcd. 573.2941]	C_32_H_42_N_2_O_6_
**CY2**	651.2007 [M+Na]^+^[calcd. 651.2046]	C_32_H_41_BrN_2_O_6_
**CY3**	565.3283 [M+H]^+^[calcd 565.3278],587.3094 [M+Na]^+^[calcd. 587.3097]	C_33_H_44_N_2_O_6_
**CY4**	565.3285 [M+H]^+^[calcd 565.3278],587.3078 [M+Na]^+^[calcd. 587.3097]	C_33_H_44_N_2_O_6_
**CY14**	607.2518 [M+Na]^+^[calcd. 607.2551]	C_32_H_41_ClN_2_O_6_
**CY15**	677.2083 [M+H]^+^[calcd 677.2088]	C_32_H_41_IN_2_O_6_
**CY18**	569.3010 [M+H]^+^[calcd 569.3027],591.2856 [M+Na]^+^[calcd. 591.2846]	C_32_H_41_FN_2_O_6_
**CY19**	603.2988 [M+Na]^+^[calcd. 603.3046]	C_33_H_44_N_2_O_7_

**m/z** = mass/charge; **calcd**. = calculated data of the compound; **MF** = Molecular Formula.

### Cyototoxicity of the Andrographolide Derivatives (Sarcosine and Proline) Series on Cancer Cell Lines Including Andrographolide

**Figure 2:**
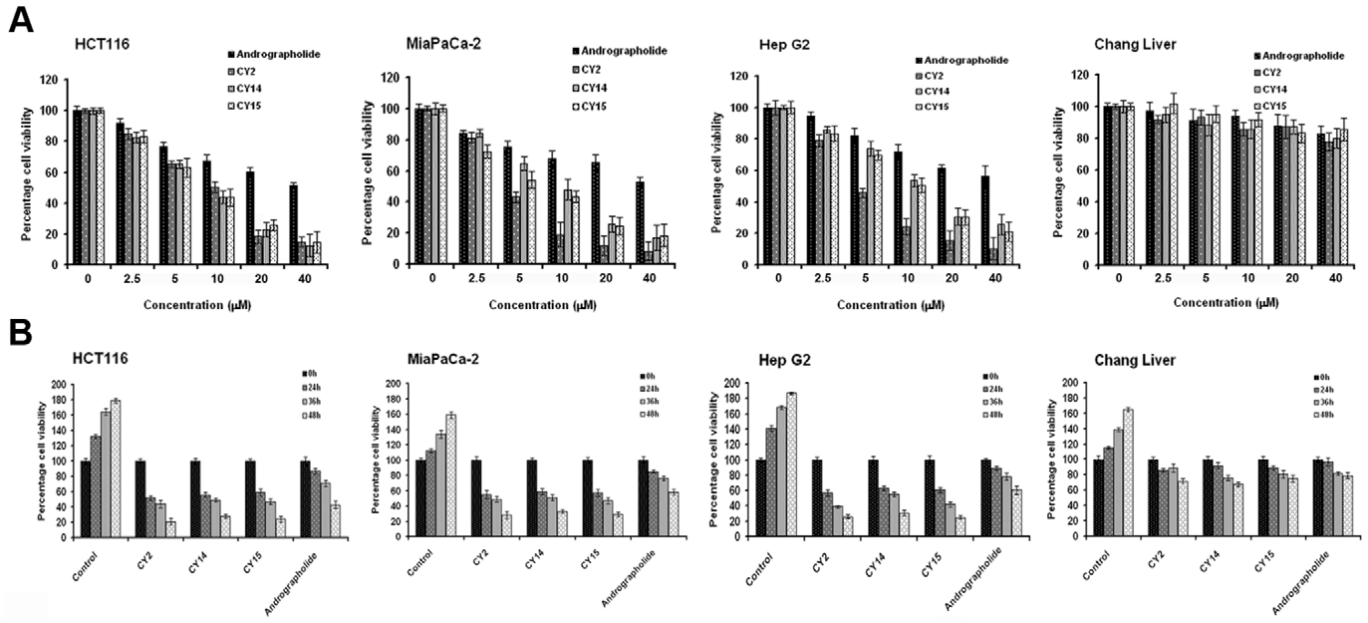
Viability of three cancer lines HCT116, MiaPaCa-2, HepG2 and a non–cancer cell line Chang liver cells was observed in response to andrographolide and its derivatives. Experimental cells (2×10^5^) were treated with andrographolide and its derivatives. MTT assay was performed. O. D. at 595 nm reflects the viability of cells. (A) Concentration dependent cell viability of potent derivatives from proline series viz. CY2, CY14, CY15 and andrographolide. (B) Time dependent cell viability of potent derivatives from proline series viz. CY2, CY14, CY15 and andrographolide. Percent viability of cells calculated from MTT assay after treatment with 20 µM of each of the potent derivatives and andrographolide for indicated time points. Values are mean ± S.D. and represent one of the 3 representative experiments (*P*<0.001).

All fifteen derivatives along with andrographolide, were examined for their cytotoxicity on a panel of six human cancer cell lines of different origins namely HCT116 (colorectal), HeLa (cervical), A375 (skin), MiaPaCa-2 (pancreatic), A549 (lung) and HepG2 (hepatic). GI_50_ values of all these compounds after 36 h treatment were shown in Table 2. As seen in the Table 2, only the GI_50_ values (for 36 h) of the halogenated di-spiropyrrolizidino oxindole (viz. proline series) derivatives (CY2, CY14, and CY15) were markedly lower than andrographolide in all the six cancer cell lines tested. However, the degree of cytotoxicity of CY2, CY14 and CY15 varied among the cell lines and they were most potent on HCT116, MiaPaCa-2 and HepG2. Effect of these potent halogenated andrographolide derivatives was dose and time dependent when a range of concentrations (0–40 µM) of these derivatives were treated and incubated for 0, 12, 24, 36 and 48 h (Fig. 2A, 2B). CY2 was the most potent derivative and its GI_50_ value on HCT116 was 10.5 µM. However, the potent derivatives did not show any cytotoxic effect on non cancer cell lines like Chang liver (Fig. 2 A, B right most panel) and peripheral blood mononuclear cells (PBMC, Fig. S6). Percent viability of 20 µM of each of CY2, CY14, and CY15 on HCT116, MiaPaCa-2 and HepG2 cells were 25–35% at 48 h and 75–80% on Chang liver cells. Similar data for 20 µM andrographolide treatment were 45–65% and 90% respectively. Similar observations were seen with trypan blue exclusion study (Fig. S5). Sarcosine, proline, azomethine ylide alone did not show any cytotoxic effect on these cells (data not shown). Thus, this study reveals that three halogenated di-spiropyrrolizidino oxindole andrographolide analogues have higher potency than andrographolide on a panel of cell lines tested here.

**Table 2:** Inhibition of HepG2 cell growth by andrographolide and its derivatives.

Compound	HCT116	MiaPaCa-2	HepG2	HeLa	A549	A375	Chang Liver
Andrographolide	>40.0	>40.0	>40.0	>40.0	>40.0	>40.0	>40.0
ProlineSeries							
CY1	38.8	>40.0	>40.0	>40.0	>40.0	>40.0	>40.0
CY2*	10.5	11.2	16.6	14.8	16.8	17.9	>40.0
CY3	>40.0	>40.0	>40.0	>40.0	>40.0	>40.0	>40.0
CY4	>40.0	>40.0	>40.0	>40.0	>40.0	>40.0	>40.0
CY14**	18.8	22.3	28.2	20.1	23.5	19.9	>40.0
CY15**	17.4	21.5	23.3	27.4	27.1	19.1	>40.0
CY18	31.8	24.2	23.6	23.8	34.1	30.8	>40.0
CY19	>40.0	>40.0	>40.0	>40.0	>40.0	>40.0	>40.0
SarcosineSeries							
CY5	>40.0	>40.0	>40.0	>40.0	>40.0	>40.0	>40.0
CY10	>40.0	>40.0	>40.0	>40.0	>40.0	>40.0	>40.0
CY11	>40.0	>40.0	>40.0	>40.0	>40.0	>40.0	>40.0
CY20	>40.0	>40.0	>40.0	>40.0	>40.0	>40.0	>40.0
CY21	>40.0	>40.0	>40.0	>40.0	>40.0	37.9	>40.0
CY22	>40.0	>40.0	>40.0	>40.0	>40.0	36.4	>40.0
CY23	>40.0	>40.0	>40.0	>40.0	>40.0	>40.0	>40.0

Cells were treated with different concentration of compounds ranging from 0–40 µM for 36 h. GI_50_ values were calculated from MTT assay.

* Derivative which is most potent;

** derivative(s) intermediately potent but more potent than andrographolide.

Values are mean ± S.D (ranging between 3–7%) and represent one of the 3 representative experiments (*P*<0.001).

### CY 2 Induce Apoptosis

**Figure 3:**
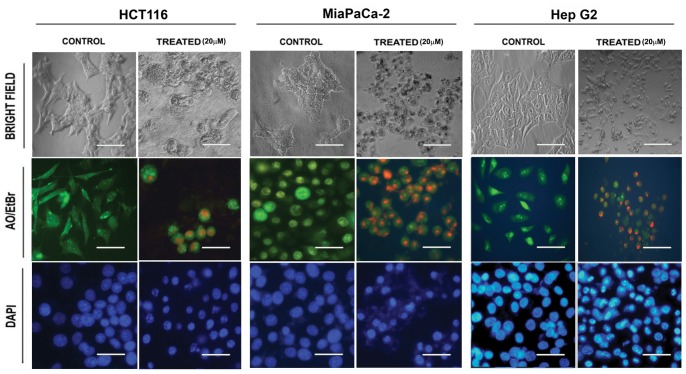
Morphological and nuclear changes seen in HCT116, MiaPaCa2 and HepG2 cells after treatment with CY2. Cells, treated with 20 µM of CY2 for 36 h were seen under light microscope and/or under fluorescence microscope following nuclear staining with DAPI or acridine orange/ethidium bromide (AO/EtBr). Results in the upper panel shows bright field, middle panel shows acridine orange/ethidium bromide (AO/EtBr) and lower panel shows DAPI stained nuclei of HCT116, MiaPaCa-2 and HepG2 cells respectively. Scale Bar = 15 µm.

Cell death induced by an anticancer agent is desired through apoptosis. As CY2 was found to be the most potent andrographolide derivatives (Table 2 and Fig. 2), involvement of apoptosis in CY2 induced cytotoxicity was examined in HCT116, MiaPaCa-2 and HepG2 cells (Fig. 3). Cell lines were taken, Light microscopic (Fig. 3, upper panel) and fluorescence microscopic study using nuclear staining dye acridine orange/ethidium bromide (AO/EtBr) and DAPI with the cells treated with 20 µM of CY2 and andrographolide for 36 h revealed characteristics apoptotic changes like higher percentage of rounded cells, condensed and fragmented nuclei unlike untreated control. Acridine orange/ethidium bromide (AO/EtBr) staining showed greenish intact nuclei in control cells, greenish-yellow, yellow, yellowish-orange, orange and/or reddish fragmented nuclei (Fig. 3, middle panel); DAPI staining showed bluish intact nuclei in control and bright fragmented nuclei (Fig. 3, lower panel) in CY2 treated cells. Thus, the microscopic study indicates that death of HCT116, MiaPaCa-2 and HepG2 cells induced by CY2 was probably due to apoptosis.

### Annexing Bound Cells Increased Following CY2 Treatment

**Figure 4:**
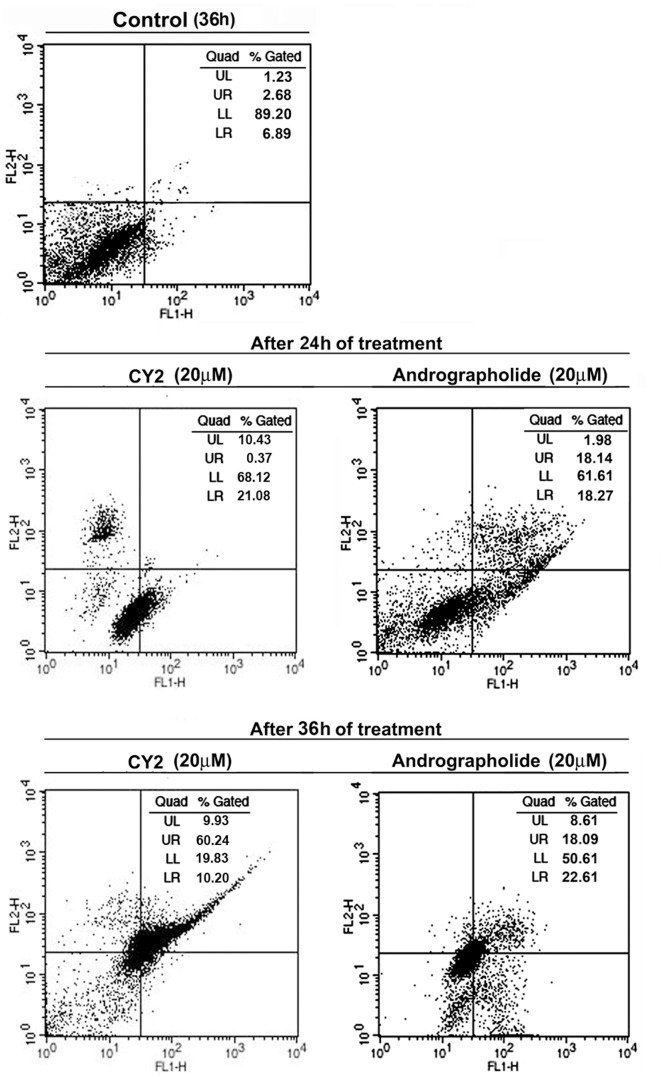
Analysis of apoptosis by flow cytometry in HCT116 cells. Cells were treated with 20 µM of CY2 (left panel) and andrographolide (right panel) for 24 h and 36 h. Binding of annexin-V/FITC to phosphatidyl serine was determined by flow cytometry. Percentages of apoptotic cells determined by the number of annexin V(+)/propidium iodide cells are shown in the scattered plot.

An important feature of apoptosis is the exposure of phosphatidyl serine from inner leaflet to outer leaflet of the plasma membrane of the cells [41], [42]. Phosphatidyl serine can bind with annexin V. This externally exposed phosphatidyl serine can be detected through flow cytometric analysis of phosphatidyl serine bound to annexin V coupled with FITC. Flow cytometric analysis revealed higher number of annexin V positive cells in CY2 treated HCT116 cells than control (Fig. 4). The percentage of viable cells was significantly low (68.12% at 24 h and 19.83% at 36 h respectively) and the total number of apoptotic cells (in LR and UR) remarkably increased following treatment of 20 µM of CY2 for 24 h (21.45%) and 36 h (70.44%). Percentage of apoptotic cells was relatively low in andrographolide treated cells (36.41% at 24 h and 40.70% at 36 h respectively). This study confirms that CY2 induces apoptosis in HCT116 cells.

Similar study in HepG2 and MiaPaCa-2 cell lines using 20 µM of CY2 revealed higher percentage of apoptotic cells than control as well as 20 µM of andrographolide treated cells (Fig. S1).

### CY2 Induces G1- Phase Cell Cycle Arrest in HCT116 Cells

**Figure 5:**
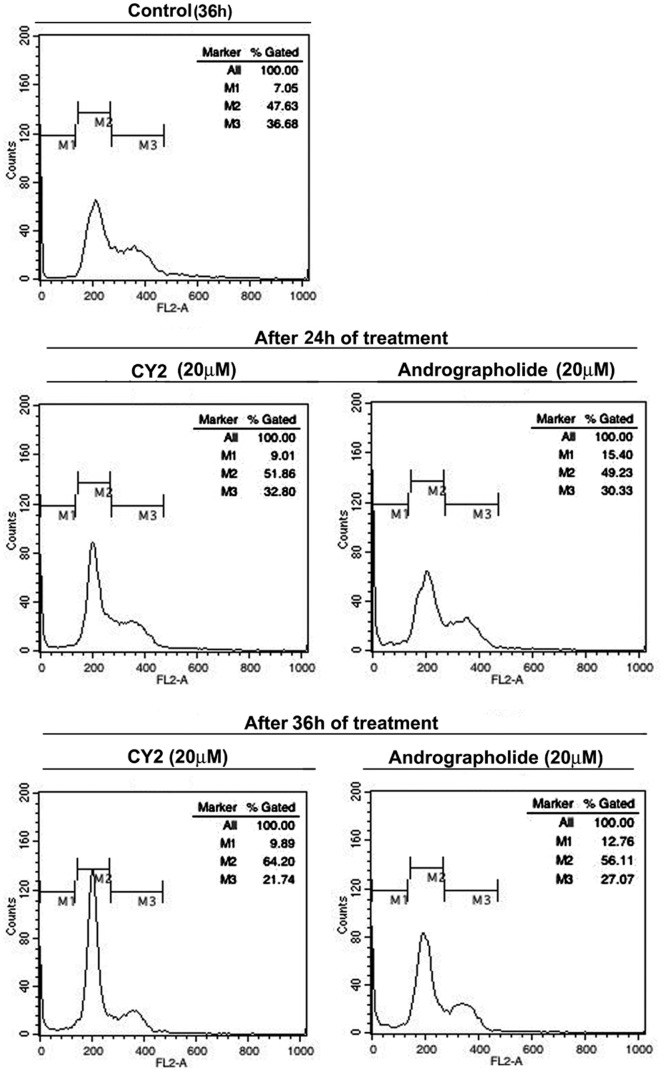
Study of cell cycle arrest in HCT116 cells by propidium iodide. Percentage of G0–G1 cell population increases after treatment of 20 µM of CY2 and andrographolide for 24 h and 36 h, indicating G1/S phase cell cycle arrest.

Apoptosis occurs through cell cycle arrest at a specific phase of cell division. Effect of CY2 on cell cycle progression was examined in HCT116 cells. As summarized in Fig. 5, treatment with 20 µM of CY2 for 24 h and 36 h showed a gradual increase in the number of cells in G1 phase. The increase in G0–G1 population for CY2 treated HCT116 cells at 24 h and 36 h in comparison with vehicle control was 4.23% and 16.57% respectively (M2 in Fig. 5). Whereas, increase in G0/G1 population for 20 µM andrographolide treatment was 1.6% at 24 h and 8.48% at 36 h. In each case, there was a concomitant reduction in the number of cells in the S and G2-M (M3 in Fig. 5) phases. Alike to HCT116 cells CY2 treated HepG2 and MiaPaCa-2 cells also showed cell cycle arrest at G1 phase (Fig. S2).

This experiment suggests that CY2 induced apoptosis involves G1-phase cell cycle arrest.

### Caspase Activation, DNA Fragmentation, Cytosolic Cytochrome c Release and Alteration in MMP

Caspases, the cysteine proteases, play a significant role to promote apoptosis by fragmenting DNA of the cell [43], [44]. Among a group of executive or effector caspases, caspase-9 is activated at the early stage in caspase dependent mitochondrial pathway of apoptosis. Activated caspase-9 then initiates the proteolytic activity of other downstream caspases, including caspase-3. DNA cleavage at the site of the protein containing structure nucleosome by caspase-3 is a key feature of apoptosis when histone proteins are exposed at the site of fragmentation [45].

**Figure 6:**
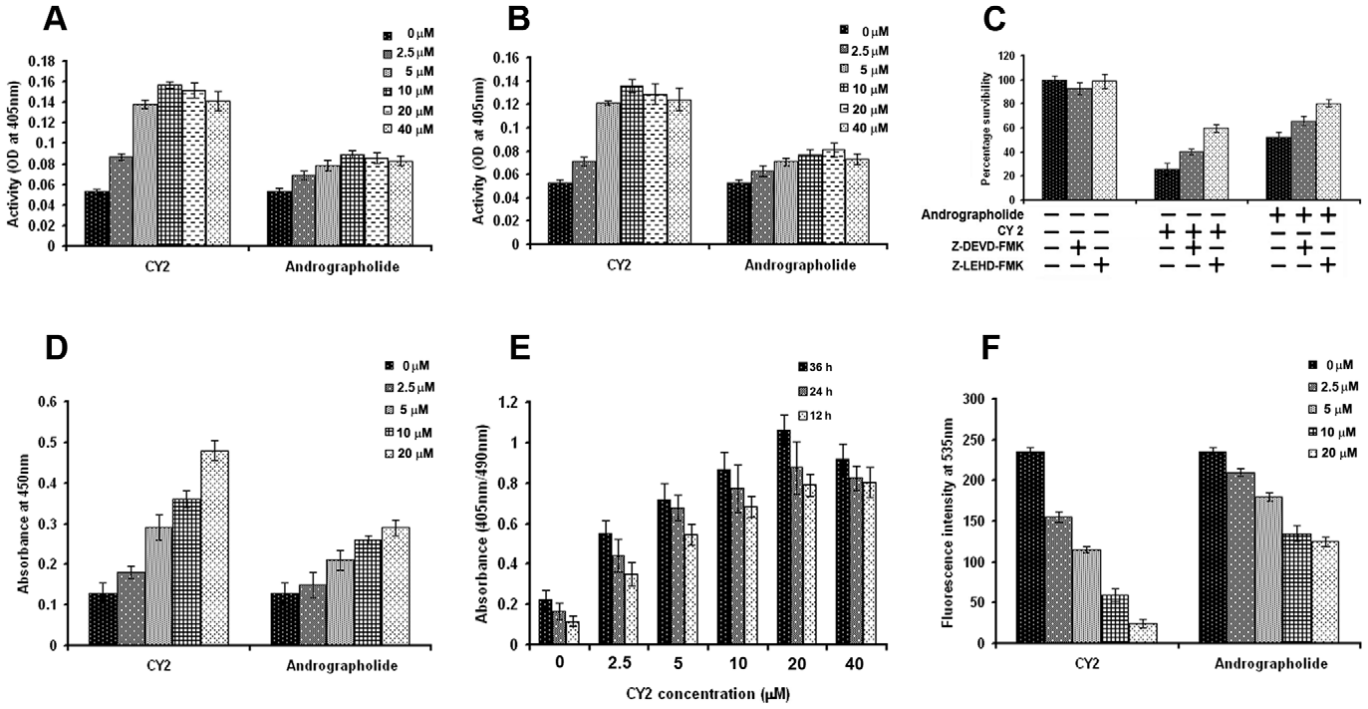
Analysis of caspase-3 and caspase-9 status, cytochrome c level, DNA fragmentation and mitochondrial membrane potential in treated and untreated HCT116 cell. Cells (2×10^5^) after treated with different concentrations (0–40 µM) of CY2 for 24 h were put to ELISA based colorimetric assay using kits for determining the levels of activated caspase-3, caspase-9, and DNA fragmentation. Enhancement of O.D. represents activation of (A) caspase-3, (B) caspase-9. (C) Cell viability in presence or absence of caspase-3 and caspase-9 inhibitors (Z-DEVD-FMK, and Z-LEHD-FMK respectively). (D) Cytochrome c level alteration determined by spectrofuorometric analysis. (E) DNA fragmentation represented through change of O.D. (F) Mitochondrial membrane potential measured by spectrofluorometry using rhodamine123 is reflected through change of O.D. Values are mean ± S.D. and represent one of the 3 representative experiments (*P*<0.001).

In the present study, status of caspase-3, caspase-9, cytosolic cytochrome c and DNA fragmentation were assessed by using respective assay kits. Caspase-3 (Fig. 6A), caspase-9 (Fig. 6B), cytosolic cytochrome c (Fig. 6D) and fragmented DNA levels (Fig. 6E) increased with increasing concentrations of CY2 as reflected with increment of their respective O. D. values. The increased levels of caspases, cytosolic cytochrome c and fragmented DNA were relatively low in andrographolide treated cells. On treatment with 20 µM of caspase-3 and caspase-9 inhibitors (Z-DEVD-FMK, and Z-LEHD-FMK respectively) the percentage of cell death induced by CY2 was lowered (Fig. 6C).

Caspase 9 activation indicates that probably apoptosis occurs via mitochondrial pathway. Loss of mitochondrial membrane potential (MMP or ΔΨm) is an essential event in mitochondrial pathway of apoptosis. MMP level was found to be lowered with treatment of the increasing concentrations of CY2 in HCT116 cells for 36 h (Fig. 6F). Disruption of MMP was relatively low in andrographolide treated cells. This, study reflects that both andrographolide and CY2 induce apoptosis of HCT116 cells occurs through caspase mediated mitochondrial pathway. Degree of disruption of MMP by CY2 was higher than andrographolide.

Treatment of 20 µM CY2 or 20 µM of andrographolide with HepG2 and MiaPaCa-2 cells resulted increase in capase-3 and -9 activation and DNA fragmentation, cytosolic cytochrome c level along with suppression of mitochondrial membrane potential (Fig. S3).

### ROS Induces DNA Damage Which Plays Role in CY 2 Induced Apoptosis

**Figure 7:**
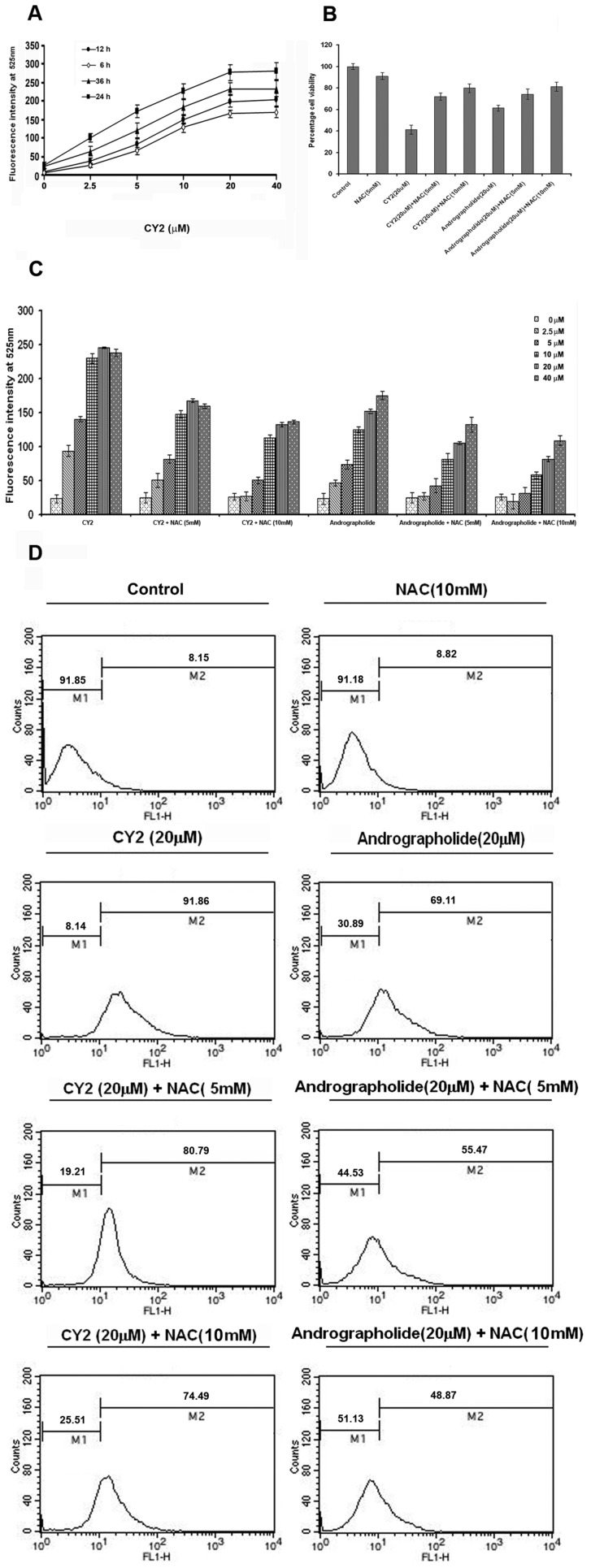
ROS generation in HCT116 cells upon treatment with 20 µM of CY2 and the scavenging action by NAC. (A) Time and dose response of ROS generation by different concentrations (0–40 µM) of CY2 incubated for 0–36 h. (B) Percentage cell viability after 20 µM CY2 and andrographolide treatment and alteration in ROS level by NAC treatment, indirectly indicating decrease in apoptotic cell death by NAC treatment. (C) Fluorometric study of ROS generation by different concentrations (0–40 µM) of CY2 and andrographolide in presence and absence of 5 mM and 10 mM NAC (D)Flow cytometric study for ROS generation by CY2 and andrographolide at 24 h on HCT116 and different degrees of scavenging effect by two concentraions (5 mM and 10 mM) of NAC. Values are mean ± S.D. and represent one of the 3 representative experiments (*P*<0.001).

Mitochondria are the major site of ROS (Reactive Oxygen Species) production in mammalian cells, and superoxide (O**^2−^**) appears to be the primary ROS produced as the result of single-electron reduction of O_2_ [46], [47]. ROS plays an important role in the induction of apoptosis in various types of cells. CY2 treated HCT116 cells produced increased amount of ROS as seen after DCF-DA treatment in fluorometric analyses. Optimum level of ROS generation was seen with 20 µM of CY2 at 24 h (Fig. 7A). Andrographolide treated cells showed maximum ROS generation at 24 h with 40 µM (data not shown). Pretreatment of 5 mM or 10 mM of NAC, an inhibitor of ROS, gradually lowered the level of cell death induced by CY2. Viability of CY2 treated HCT116 cells increased by 37.65% and 46.41% after pre-treatment with 5 mM and 10 mM NAC respectively. For 20 µM of andrographolide, same values were 20.15% and 27.79% respectively (Fig. 7B). It indicates that cell viability increases by 8.76% and 7.65% after increasing the NAC concentration from 5 mM to 10 mM followed by 20 µM of CY2 and andrographolide treatment respectively. ROS generation increased with increasing concentrations (0–40 µM) of CY2 and andrographolide which was inhibited by NAC (Fig. 7C). Changes in ROS generation in flow cytometric analyses was similar to that of fluorometric assays (Fig. 7D). In CY2 and andrographolide treated cells pre-incubated with increasing concentrations of NAC, percent cell death was found to be lowered gradually. The ratio of DCF-positive cells in flowcytometric analysis was 8.15% for vehicle control and 8.82% for 10 mM NAC treated cells at 24 h. This ratio was 91.86% for 20 µM of CY2 treated cells at 24 h and decreased to 80.79% and 74.49% with 5 mM and 10 mM NAC pretreated cells respectively. This reveals that, ROS generation decreased by 2.11 and 2.82 fold for increasing the NAC concentration from 5 mM to 10 mM. On 20 µM andrographolide treatment for 24 h, the ratio was 69.11% which decreased to 55.47% and 48.87% in cells pre-incubated with 5 mM and 10 mM NAC. This indicates that ROS generation is decreased by 2.19 and 2.94 fold when pre-incubated with 5 mM and 10 mM NAC (Fig. 7D). Fluorometric and flow cytometric results indicate that CY2 induced apoptosis of HCT116 cells is ROS dependent. Similar fluorometric study in HepG2 and MiaPaCa-2 cell lines using 20 µM of CY2 revealed higher level of ROS generation than control as well as 20 µM of andrographolide treated cells after 24 h. ROS generation decreased in both the cell lines when pre-incubated with 10 mM NAC (Fig. S4).

### CY 2 Alters the Level of Bcl-2 Family Proteins, P53, Caspases, PARP,NF-κB p65, Akt, p-Akt, PI3k and MMP-9 in HCT116 Cells

**Figure 8:**
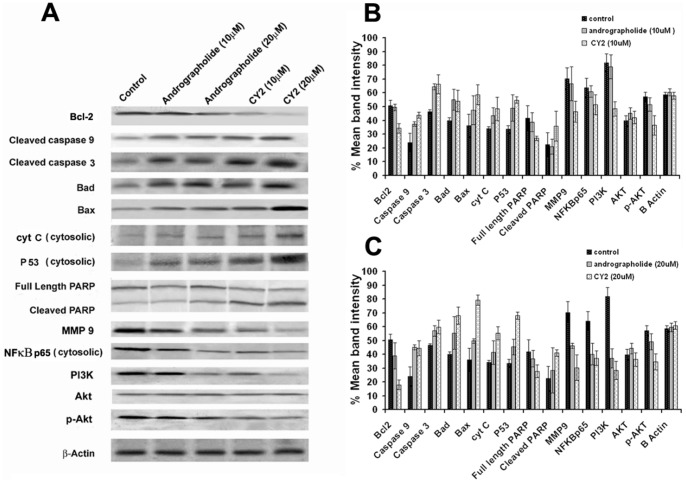
Western Blot analyses of some important pro- and anti-apoptotic proteins in treated and untreated HCT116 cells. Cells were treated with 10 µM or 20 µM each of CY2, CY14, CY15 for 24 h or left as such. Cells were lysed and cell lysate were used for Western Blot analysis. (A) Panel represents western blot analysis of different apoptotic proteins. Panel indicates Bcl2, Bad, Bax, caspase 3 and 9, cytochrome c, P53,PARP, NF-κB p65 (cytosolic), PI3K, Akt, p-Akt and β-Actin expression after 10 µM or 20 µM of andrographolide and CY2 treatment respectively for 24 h. (B) Bar graph shows semiquantified densitometry from western blot analysis for lower dose (10 µM) of both andrographolide and CY2 treatment. (C) Bar graph shows semiquantified densitometry from Western blot analysis for higher dose (20 µM) of both andrographolide and CY2 treatment (*P*<0.001).

Cellular apoptosis mediated via mitochondrial pathway is dominated by a number of pro- and anti-apoptotic protein families such as the anti-apoptotic Bcl-2 subfamily and the pro-apoptotic Bax subfamily proteins [48], [49]. Bax of the Bcl-2 family member proteins governs the release of cytochrome c from mitochondria to cytosol. Cytochrome c released from the mitochondria in response to apoptotic stimuli activates procaspase-9 at the early stage of apoptosis. Caspase-9 in turn activates caspase-3 and PARP cleavage, a nuclear protein that detects DNA strand breaks and functions in base excision repair. Proteolytic cleavage of PARP by caspases, mainly by caspase-3 is a hall mark of apoptosis. Once PARP is cleaved, it no longer supports the enzymatic DNA repair function. The tumor suppressor gene p53 is responsible for triggering the apoptosis process within the cells. Along with P53, other important mediators of apoptosis are PI3K, p-Akt, NF-κB p65 and MMP-9. Western blot analysis revealed that the treatment of HCT116 cells with CY2 and andrographolide lowered the levels of Bcl-2 whereas level of Bax, and Bad increased (Fig. 8A). Caspase-3 and caspase-9 were enhanced with increasing concentration of the derivatives. Cytosolic cytochrome c and cleaved PARP level also increased in parallel in the treated cells compared to untreated control. Western blot data showed upregulation of p53 along with the downregulation of PI3K, p-Akt, NF–κB p65 and MMP-9 proteins. Densitometric analysis of the western blot data was shown in Fig. 8B and 8C.Thus, these data reveal that CY2, induced apoptosis of HCT116 cells proceeds via mitochondrial pathway of apoptosis along with alteration of some important apoptosis regulatory proteins.

### Translocation and Localization of P53/P21, NF-κB p50/p65 and MMP-9

Western blot data (Fig. 8A) showed upregulation of p53 along with the downregulation of p-Akt, NF-κB p65, PI3K, and MMP-9 proteins which are important mediators of cell cycle arrest. So our endeavor was to investigate their cellular localization in CY2 treated and untreated HCT116 cells.

**Figure 9:**
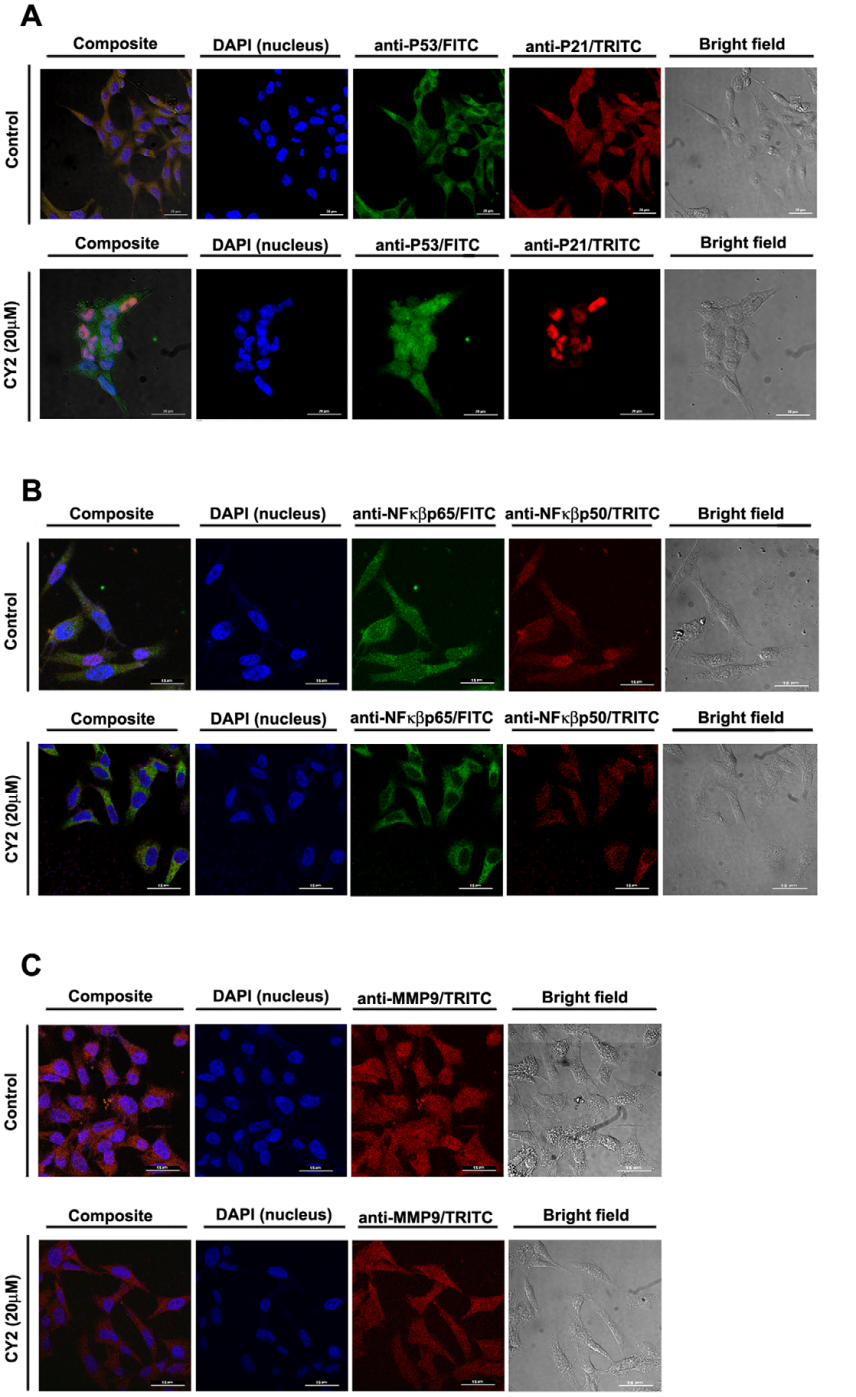
Immunocytochemistry showing localization of P53, P21, NF-κB p50, NF-κB p65 and MMP-9 in presence and absence of CY2 in HCT116 cells. DAPI was used for genomic DNA counterstaining (A) Translocation status of P53 and P21 in control cells (upper panel) and 20 µM CY2 treated cells (lower panel). HCT116 cells coincubated with primary antibodies for P53 and P21 and developed with FITC (λ_Em_∼525 nm, green) and TRITC (λ_Em_∼576 nm, red) labeled secondary antibodies respectively, cotranslocation (left composite panel) in the nuclei was observed for both of these proteins with respect to the untreated cells, where they were found to reside mainly in the cytoplasm. Scale Bar = 20 µm. (B) Translocation status of NF-κB p50 and NF-κB p65 in control cells (upper panel) and 20 µ M CY2 treated cells (lower panel). The nuclear localization of the two main subunits of the NF-κB family viz. p65 and p50 (developed with FITC and TRITC -labeled secondary antibodies respectively) was found in untreated control cells but upon treatment, these two subunits tends to localize into the cytoplasm with respect to the untreated control as shown in the composite confocal micrograph. Scale Bar = 15 µm. (C) Localization of MMP-9 in control cells (upper panel) and 20 µM CY2 treated cells (lower panel). Confocal microscopy revealed that immunocytochemistry of MMP-9 protein (developed with TRITC-labelled secondary antibody) expression decreased following treatment with CY2 with respect to the untreated control. Scale Bar = 15 µm.

CY2 treated (20 µM) and untreated HCT116 cells were incubated for 24 h with primary antibodies raised against proteins such as P53, P21, MMP-9, NF-κB p65 and p50 subunits (Fig. 9). DAPI was used for genomic DNA counterstain. In case of cells coincubated with primary antibodies for P53 and P21 and developed with FITC (λ_Em_∼525 nm) and TRITC (λ_Em_∼576 nm) labeled secondary antibodies respectively, cotranslocation (left composite panel of Fig. 9A) in the nuclei was observed for both of these proteins with respect to the untreated cells, where they were found to reside mainly in the cytoplasm (Fig. 9A).

The nuclear localization of the two main subunits of the NF-κB family viz. p65 and p50 (developed with FITC and TRITC-labeled secondary antibodies respectively) was found in untreated control cells but upon treatment, these two subunits tends to localize into the cytoplasm with respect to the untreated control as shown in the composite confocal micrograph (Fig. 9B), thereby showing a lack of cell viability upon treatment.

Confocal microscopy revealed that immunocytochemistry of MMP-9 protein (developed with TRITC-labeled secondary antibody) expression decreased following treatment with CY2 with respect to the untreated control (Fig. 9C).

## Discussion

Andrographolide (3-[2-[decahydro-6-hydroxy-5-(hydroxymethyl)-5, 8a-methyl-2-methylene-1-napthalenyl] ethylidene] dihydro-4-hydroxy-2(3H)-furanone), the major labdane diterpene constituent of *Andrographis paniculata* of Acanthaceae family, is used extensively in the traditional system of medicine in Southeast Asia and other parts of the world since antiquity [50], [51]. Andrographolide has well reported anticancer and ant-inflammatory properties. Synthesis of more effective derivatives from andrographolide has been pursued *in lieu* of finding better drug candidate than andrographolide. Therefore, in the present study, the cytotoxic efficacy of different di-spiropyrrolidino and di-spiropyrrolizidino oxindole andrographolide analogues prepared by cycloaddition of azomethine ylide and sarcosine or proline (called here sarcosine and proline series respectively) at 12, 13 double bond of andrographolide, were evaluated. Fifteen di-spiropyrrolidino and di-spyropyrrolizidino oxindole analogues of andrographolide using HCT116 and five other carcinoma cell lines, viz. MiaPaCa-2, HepG2, HeLa, A375 and A549 reveals that only three compounds CY2, CY14, and CY15 are much more potent than the andrographolide itself. These are di-spiropyrrolizidino oxindole (proline series) derivatives, having Br, Cl, or I groups in the aromatic ring. Other compounds of this series having –H, –CH_3_, or –OCH_3_ groups and the sole azomethine ylide analogue(s) showed less cytotoxicity than CY2, CY14 and CY15. Compounds of di-spiropyrrolidino oxindole (sarcosine series) derivatives with –H, –CH_3_, –OCH_3_ or even halogen groups did not show better cytotoxic activity than andrographolide. Since the chemical reactivity of halogen atoms depends on both their point of attachment to the lead and the nature of the halogen [52] Thus, it can be concluded that higher activity of CY2, CY14, and CY15 may be due to the combined effect of the di-spiropyrrolizidino oxindole ring attached at the double bond between C-12 and C-13 position of andrographolide and the Br, Cl or I group. However among the potent three analogues, bromo-derivative (CY2) of andrographalide showed highest cytotoxic potential and needed to be elucidated further. Very limited reports are there on the anticancer activity of andrographolide derivatives. There are quite a few reports regarding higher cytotoxic potencies of various andrographolide derivatives in comparison with their parent compound (andrographolide) towards various cancer cells. Nanduri et al (2004), had observed the higher potencies of the ester derivatives of 8, 17-epoxy andrographolide analogues towards breast (MCF-7/ADR), colon (SW 620), CNS (U251), lung (H522), ovarian (SKOV3), prostrate (DU145) and renal (A498) when compared to the potency exerted towards the same cells by the parent compound, andrographolide. Satyanarayana *et al.* (2004) compared the cytotoxic potential of andrographolide and its semi synthetic analogue, DRF3188 towards MCF-7 breast cancer cells. Both these compounds inhibited cell cycle arrest at G0–G1 phase by up regulation of p27 and down regulation of Cdk4 at a concentration of 25 µM and 5 µM respectively. Hai-Wei Xu *et al.* (2006), observed higher cytotoxic potencies of andrographolide analogues with respect to the parent compound andrographolide towards CNE and ECa 109 human cancer cell lines. Jada *et al.* (2006) had observed higher cytotoxicities against MCF-7(breast) and HCT115 (colon) cancer cells when compared with the potency of the parent andrographolide towards the same cell lines. Also, Jada *et al.* (2007) observed that andrographolide analogues, 3, 19 isopropylideneandrographolide was effective against leukemia and colon cancer cell lines whereas, 14 acetylandrographolide was effective against leukemia, ovarian and renal cancer cells in a dose dependent manner. Both these compounds exhibited non specific cell cycle arrests both in a dose and time dependent manner. Only the benzylidene derivatives synthesized by coupling the two hydroxyl groups at C-3 and C-19 through substitution of aromatic aldehydes has *hitherto* examined for anticancer activity. Similarly, the same group (Jada *et al.* 2008) observed higher potency for andrographolide derivatives such as 3, 19-(2-bromobenzylidene) andrographolide (SRJ09) and 3, 19-(3-chloro-4-fluorobenzylidene) andrographolide (SRJ23) than andrographolide. SRJ09 and SRJ23 induced G1 cell cycle arrest and apoptosis in MCF-7 and HCT-116 cells, respectively. One of the benzylidene derivatives is shown to be the lead compound with better activity than andrographolide. Finally, Das *et al.* (2010) observed higher potencies for andrographolide analogues prepared through chemo-selective functionalization at C14 hydroxy towards various human leukemic cell lines when compared to the parent compound andrographolide. Moreover, andrographolide along with its analogues showed less cytotoxicity towards normal cell lines, L132 and NIH3T3, when compared to the cancer cells [12], [53]–[58].

Halogenated natural products are rare in nature. Our study shows that chlorinated and brominated andrographolide derivatives of proline series are highly potential than fluorinated or iodinated derivatives. This may be due to cumulative effect resulted from electronegativity and size of the halogenated derivatives. Bromine occupies a central position on the halogen series. Bromine is being larger in size and also moderate halogen bond acceptor than fluorine. The C-Br bond is enough stable, allowing its insertion on diverse heterocyclics of pharmacological value. Another feature of brominated drugs regards to its binding affinity for some cellular proteins. Replacing hydrogen by bromine also provides a substantial alteration on the volumetric and shape issues. The subunits bearing bromine can be accommodated in tight and deep cavities, as well as in hydrophobic pockets of the biological targets [59]. Chlorine occupies an intermediate position in between fluorine and bromine. Chlorine is a better halogen bond acceptor, besides being smaller in size than bromine. The C-Cl bond is also enough stable, allowing its insertion on diverse heterocyclics of pharmacological value similar to that of bromine.

From our study, three halogenated andrographolide derivatives have been identified to show potent cytotoxic activity than andrographolide. Halogens are important functional groups. Number of halogenated anticancer drugs is less compared to the derivatives having other functional groups. Binary protein-halogenated ligand complexes are more stable than non-halogenated ligands because of favorable electrostatic interactions of the halogen bonds. Cyclin-dependent kinase 2 (CDK2) plays critical roles in important intracellular pathways and cell cycle progression, being thus an attractive biological target for drug development [60]–[62]. Auffinger and co-workers showed that halogenated ligands bind to the cyclin-dependent kinase 2 (CDK2), leading to more stable complexes than non-halogenated ligands [63]. The incorporation of halogen atoms into new bioactive chemical entity is commonly used to increase membrane permeability and therefore, improve the oral absorption [64]. Halogenation also enhances the blood brain barrier (BBB) permeability and this is a pre-requisite for many drugs [65]. In this respect our study of new halogenated anticancer agents are important findings and adds a new approach in cancer research.

It is believed that carbon-halogen bonds are not easily metabolized by the cytochrome p450 system and, therefore, it is a feasible strategy to block the metabolically labile positions of a particular new bioactive chemical entity [66] that validates our results.

An important feature of these lead derivatives is that the cell death triggered by them involves apoptosis as seen in HCT116 cells of our study. Typical features of apoptosis are cell rounding, nuclear condensation and DNA fragmentation and externalization of phosphatidyl serine were observed in response to the treatment of CY2, CY14, and CY15. Apoptosis induced by these three derivatives in HCT116 cells were found to be mediated via mitochondrial pathway with loss of mitochondrial membrane potential, caspase-3 and caspase-9 activation, PARP cleavage, increase of cytosolic cytochrome c level and alteration of Bcl-2, Bax and Bad protein levels. Zhou *et al.* has shown that andrographolide induced apoptosis in HCT116 cells was associated with loss of mitochondrial potential and activation of caspae-3, but were not affected by caspase inhibitors [67]. Andrographolide induced apoptosis in HCT116 cells is followed by alteration of Bcl-2/Bax ratio, activation of Bad, p53 and caspase-3 [48]. Our study shows that CY2, CY14, and CY15 induced apoptosis in HCT116 cells proceeds via mitochondrial pathway in caspase dependent manner (Fig. 6). The data suggests that the mode of action of the derivatives on HCT116 cells is similar to that of andrographolide. Change in mitochondrial membrane potential lead us to investigate the process of apoptotic cell death via ROS generation. Potent derivative and andrographolide both induced ROS mediated apoptotic cell death which was inhibited by NAC as evidenced by flow cytometric and fluorometric assay.

Another interesting point of our study is that CY2, CY14, and CY15 are more potent inducer of apoptosis than andrographolide and do not show any cytotoxicity at the doses used in human peripheral blood mononuclear cells (PBMC) as well as Chang liver cells. Thereby, CY2, CY14, and CY15 may be regarded as potential anticancer candidates.

Andrographolide induces apoptosis via inhibiting NF-κB induced bcl-2 mediated survival signaling and modulating p53 induced caspase-3 mediated proapoptotic signaling. Andrographolide has been reported to block the activation and translocation of NF-κB subunits such as p65, p50 and c-Rel to the nucleus; also inhibits angiogenesis by inhibiting by inhibiting MMP-2 and MMP-9. Andrographolide induces apoptosis in B16F-10 melanoma cells by inhibiting NF-κB mediated bcl-2 activation and modulating p53-induced caspase-3 gene expression [68]. Inhibitory effects of andrographolide on migration and invasion in human non-small cell lung cancer A549 cells via down-regulation of PI3K/Akt signaling pathway [6]. CY2 induced apoptosis of HCT116 cells is associated with inhibition of NF-κB (p65, p50) translocation and upregulation of P53 and P21, downregulation of MMP-9 and PI3K. CY2 which showed higher potency than andrographolide (as shown earlier), shares the same mechanism of inducing apoptosis of the latter. In conclusion, from our study, three halogenated andrographolide derivatives have been indentified to show potent anticancer activity than andrographolide. Halogens are important functional groups but number of halogenated drugs is less, compared to the other functional groups as anticancer agents. In this respect, our findings are important to explore anticancer agent(s). Probably, biological potency of the derivatives results from the combined effect of the nature of halogens and the structures of the compounds where it is attached. In addition, we can conclude that the potent compound CY2 although is structurally different from andrographolide to some extent, its mode of functions towards apoptosis induction in cancer cells may be similar to that of andrographolide.

## Materials and Methods

### Reagents

Dulbecco’s modified Eagle medium (DMEM), fetal bovine serum (FBS), penicillin, streptomycin, neomycin (PSN) antibiotic, trypsin and ethylene di-amine tetra-acetic acid (EDTA) were obtained from Gibco BRL (Grand Island, NY, USA). Tissue culture plastic wares were obtained from NUNC (Roskilde, Denmark). Rabbit anti-Bcl2, Bad, Bax, caspase 3 and 9, cytochrome c, P53, PARP, NF-κB p65, and β-Actin and Mouse anti-P21 and NF κB/p50 polyclonal antibodies were obtained from Santa Cruz Biotechnology (Santa Cruz, CA). Rabbit anti-PI3K, Akt, p-Akt polyclonal antibodies were obtained from Cell Signaling Technology. Organic solvents used were of HPLC grade. All other chemicals including doxorubicin, NAC used were from Sigma Chem. Co. (St. Louis, MO, USA) or mentioned otherwise.

### Preparation of Andrographolide Derivatives

Di-spiropyrrolidino and di-spiropyrrolizidino oxindole derivatives of andrographolide were prepared by Hazra *et al.* [13].

### Cell Culture

Cell lines used here were obtained from National Centre for Cell Science, Pune, India. These cell lines were cultured in DMEM or RPMI 1640 supplemented with 10% FBS and 1% antibiotic (PSN), 5 µg ml^−1^ of Plasmocin ™ Prophyllactic (Invivogen, San Diago, USA) and at 37°C in a humidified atmosphere with 5% CO_2._ Regular monitoring of culture by staining with DAPI and Hoechst 33258 (Sigma Aldrich, India) is done and found no small dots or flecks of fluorescence concentrated on the cell surface and in the surrounding medium and on the culture dish as the indicator of presence of mycoplasma DNA. After achieving 75–80% confluence, cells were harvested with 0.025% trypsin and 0.52 mM EDTA in phosphate buffered saline (PBS) and were seeded at desired density to allow them to re-equilibrate a day before the start of experimentation. All experiments were conducted in DMEM supplemented with 10% FBS and 1% antibiotic (PSN) solution.

### Cell Viability Assay

MTT [3-(4, 5-Dimethylthiazol-2-yl)-2, 5-diphenyltetrazolium bromide] assay was used to evaluate cell viability as previously described [69]. In each well of a 96-well culture plate 2×10^5^ cells were seeded in triplicate. Briefly, mono-layers of cells were treated with or without andrographolide or its analogues at concentrations ranging from 0–40 µM for 0–48 h. At the end of the culture period, 20 µl of 5 mg ml^−1^ MTT stock solution was added in each well. After additional 4 h incubation at 37°C, the resultant intracellular formazan crystals were solubilized with acidic isopropanol and the absorbance of the solution was measured at 595 nm using an ELISA reader (Model: Emax, Molecular device, USA). Absorbance (O.D.) of medium containing the different concentrations of the compounds (0–40 µM) and MTT reagents was subtracted from the respective experimental sets and then percent viability was calculated.

### Determination of Cytotoxicity

Trypan blue exclusion assay was performed for cytotoxicity determination [70]. Briefly, the cells were plated at a density of 1×10^5^ in 24-well flat-bottomed tissue culture (TC) plates and either treated with andrographolide or its derivatives at concentrations ranging from 0–40 µM for 36 h or left on such untreated. Those cells were then harvested, washed twice with PBS, concentrated to 200 µl and stained with 0.4% trypan blue. Approximately 100 cells were counted with a hemocytometer for each experiment. The percentage of cytotoxicity was calculated as follows:

% Cytotoxicity = (trypan blue positive cells/total cells counted) × 100.

### Assessment of Cell Morphology

Cells (3×10^4^/well) were grown in 6-well TC plates and treated with or without andrographolide or its derivatives at concentrations ranging from 20 µM for 36 h. Morphological changes were observed with an inverted phase contrast microscope (Model: OLYMPUS IX70, Olympus Optical Co. Ltd., Shibuya-ku, Tokyo, Japan) and photographs were taken with the help of a digital camera (Olympus, Inc. Japan).

### Fluorescence Microscopy

To detect nuclear damage or chromatin condensation, treated and untreated cells were washed twice with PBS and fixed with 3.7% paraformaldehyde at room temperature for 2 h. Fixed cells were stained with 10 µg ml^−1^ of 4′, 6′-diamidino-2-phenylindole (DAPI) and observed under fluorescence microscope (Model: OLYMPUS IX70, Olympus Optical Co. Ltd., Shibuya-ku, Tokyo, Japan) with excitation at 359 nm and emission at 461 nm [71]. The conventional acridine orange/ethidium bromide (AO/EtBr) staining procedure was followed to differentiate the live, apoptotic and necrotic cells [72]. Briefly, treated or untreated cells were stained with acridine orange (50 µg ml^−1^) and ethidium bromide (50 µg ml^−1^) and analyzed under fluorescence microscope with LASER beam excitation at 488 nm and 550 nm. Photographs were acquired.

### Quantification of Apoptotic Cell Death

Apoptosis was assayed by using an annexin V-FITC apoptosis detection kit (Calbiochem, Germany). Cells treated with or without andrographolide or its derivatives, were stained with PI and annexin V- FITC according to manufacturer’s instructions. The percentage of live, apoptotic and necrotic cells were analyzed by Flow Cytometry immediately (Becton Dickinson, San Jose, CA, USA). Data from 10^6^ cells was analyzed for each sample. Spectrophotometry at 405 nm with ELISA based cell death detection kits (Calbiochem, Germany) were used to detect apoptotic cell death by measuring the level of DNA fragmentation in the lysates of cells untreated or treated with andrographolide or its analogues.

### Cell Cycle Arrest Assay

Subconfluent cells were treated with CY2 and andrographolide (0 and 20 µM) in culture medium as described above for 24 h and 36 h. The cells were then harvested, washed with cold PBS, and processed for cell cycle analysis. Briefly, 1×10^5^ cells were resuspended in 50 µl of cold PBS, to which cold methanol (450 µl) was added, and the cells were then incubated for 1 h at 4°C. After centrifugation, the pellet was washed with cold PBS, suspended in 500 µl PBS, and incubated with 5 µl RNase (20 µg ml^−1^ final concentration) for 30 min. The cells were kept on ice for 10 min and incubated with propidium iodide (50 µg ml^−1^ final concentration) for 1 h in the dark [73], [74]. The cell cycle distribution of the cells of each sample was then determined using a FACSCalibur instrument (BD Biosciences, San Jose, CA) equipped with CellQuest 3.3 software. ModFit LT cell cycle analysis software was used to determine the percentage of cells in the different phases of the cell cycle. All aggregates were removed during analysis by an appropriate gate on Area/Width parameters and DNA content analysis was performed on ≥10,000 gated cells.

### Caspases and Cytochrome c Assay

Levels of caspase-3, caspase-9 and cytochrome c in the lysates of treated or untreated cells were assayed by using ELISA based colorimetric assay kits of caspase-3,caspase-9 (Santacruz Biotechnology, Germany/USA) and cytochrome c assay kit (Invitrogen Corporation, Camarillo, CA USA) respectively. Caspase 3 inhibitor Z-DEVD-FMK and caspase 9 inhibitor Z-LEHD-FMK were bought from R&D Systems, Inc. McKinley Place NE Minneapolis, MN USA).

### Measurement of Mitochondrial Membrane Potential

To measure mitochondrial membrane potential (MMP), both treated or untreated cells were washed twice with ice-cold PBS before incubation with rhodamine123 (5 µg ml^−1^) in darkness for 15 min at room temperature (37°C). Emission was measured in a spectrofluorometer (LS50B; PerkinElmer) using fluorescent intensity at 535 nm.

### Measurement of ROS Generation

ROS generation was measured by DCF-DA. After treatment with 25 µM CY2 for the indicated time periods, the cells were incubated with 10 mM DCF-DA at 37°C for 15 min. The intracellular reactive oxygen species (ROS) mediated oxidation of DCF-DA to the fluorescent compound 2′, 7′-dichlorofluorescein (DCF). Then cells were harvested and the pellets were suspended in 1 ml PBS. Samples were analyzed at an excitation wave length of 480 nm and an emission wave length of 525 nm by both FACSCalibur flowcytometry (Becton Dickinson, San Jose, CA, USA) and by spectrofluorometer.

### Western Blot Analysis

To determine protein expression levels, Western blot analysis was performed by the technique described earlier with minor modifications [75]. Briefly, with either treated or untreated cells both adherent and floating cells were collected and lysed. Lysates (40 µg of protein) were separated by electrophoresis in 10% SDS-polyacrylamide gel and electro-transferred to PVDF membranes (Immobilon-P, Millipore Corporation, Bedford, MA, USA) using a trans-blot system (Transblot SD: Semidry transfer cell; Bio-Rad Laboratories, Inc., Hercules, CA, USA). The membranes were blocked with 5% non-fat dry milk in TBST (Tris Buffered Saline containing 0.1% Tween-20, pH 7.6) for 1 h at room temperature and then probed with primary antibodies overnight at 4°C. Rabbit anti-Bcl2, Bad, Bax, caspase 3 and 9, cytochrome c, P53, PARP, NF-κB p65, and β-Actin and mouse NF-κB p50 polyclonal antibodies were obtained from Santa Cruz Biotechnology (Santa Cruz, CA). Rabbit anti-PI3K, Akt, p-Akt polyclonal antibodies were obtained from Cell Signaling Technology. After extensive washes membranes were incubated with alkaline peroxidase conjugated secondary antibody for 1 h. Immuno-reactive bands were visualized by adding NBT/BCIP. Loading control was assessed using antibodies to β-Actin.

### Confocal Laser Scanning Microscopy for Immunocytochemistry Study

For immunocytochemical analysis of cell cultures, cells were seeded onto glass coverslips at a density of 100,000 cells/cm^2^, fixed with 4% paraformaldehyde in PBS for 20 min, permeabilized with 0.1% Triton X-100 in PBS for 20 min, and exposed to blocking solution (PBS containing 5% bovine serum albumin and 0.2% Tween 20) for 1 h at room temperature [76]. Cultures were then incubated with primary antibodies [viz. rabbit anti-P53, MMP-9, NF-κB p65; mouse anti-NF-κB p50 (Santa Cruz), P21 (Cell signaling) polyclonal antibody] and developed with TRITC or FITC-labeled secondary antibody. All primary antibodies were used at 1∶100 dilutions for two hours at room temperature. After washing several times with PBS, cells were stained for two hours at room temperature, with secondary antibody fluorescently labeled in green or red, specific for mouse, rabbit or goat and diluted 1∶300. DAPI was used for genomic DNA counterstaining. Control slides were treated the same way except that primary antibodies were omitted, resulting in no visible staining. Samples were mounted in Vectashield hard set mounting media (Vector Laboratories) and imaged on a Nikon Eclipse C1 confocal microscope.

### Statistical Analysis

All values were reported as mean ± SD and Student’s t test was used for determining statistical significance (P<0.001).

## Supporting Information

Figure S1 **Analysis of apoptosis by flow cytometry in HepG2 and MiaPaCa-2 annexin-V/FITC.** Cells were treated with 20 µM of CY2 (left panel) and andrographolide (right panel) for 24 h. Binding of annexin V to phosphatidyl serine was determined by flow cytometry. Percentages of apoptotic cells determined by the number of annexin V (+)/propidium iodide cells are shown in the scattered plot. (A) Apoptosis analysis in HepG2 cells. (B) Apoptosis analysis in MiaPaCa-2 cells.
(TIF)

Figure S2 **Study of cell cycle arrest by propidium iodide in HepG2 and MiaPaCa-2.** Cells treated with or without 20 µM of CY2 and andrographolide for 24 h were used for cell cycle arrest study as described in materials and methods. Percentage of G0–G1 cell population increases after treatment of indicating G1/S phase cell cycle arrest. (A) Cell cycle arrest in HepG2 cells. (B) Cell cycle arrest in cells MiaPaCa-2.
(TIF)

Figure S3 **Analysis of caspase-3 and caspase-9, DNA fragmentation, mitochondrial membrane potential and cytochrome c level Cells (2×10^5^) after treated with 20 µM of CY2 for 24 h by ELISA based colorimetric assay using kits in HepG2 and MiaPaCa2.** Enhancement of O.D. represents activation of (A) caspase-3, (B) caspase-9. (C) DNA fragmentation (D) Mitochondrial Membrane Potential (E) Cytochrome c level in HepG2 and MiaPaCa-2 cell lines. Values are mean ± S.D. and represent one of the 3 representative experiments (*P*<0.001).
(TIF)

Figure S4 **A relative ROS generation by 20 µM of CY2 and andrographolide with the scavenging action by NAC in HepG2 and MiaPaCa-2 cell lines.** Flourosence intensity of 2′,7′-dichlorofluorescein (DCF) in HepG2 and MiaPaCa-2 cell lines after treatment of 20 µM of CY2 and andrographolide incubated for 24 h in treated without NAC and NAC (Values are mean ± S.D. and represent one of the 3 representative experiments (*P*<0.001).
(TIF)

Figure S5 **Viability of three cancer lines HCT116, MiaPaCa-2, HepG2 and a non–cancer cell line Chang liver cells was observed in response to andrographolide and its derivatives.** Experimental cells (2×10^5^) were treated with andrographolide and its derivatives. Assay of dead cells by trypan blue exclusion test. Cells, untreated or treated with different concentrations of andrographolide and its derivatives of sarcosine and proline series for 36 h, were stained with trypan blue. Stained (dead) cells were counted under microscope. Number of dead cells is presented following treatment of derivatives of CY2, CY14, CY15 and andrographolide. Values are mean ± S.D. and represent one of the 3 representative experiments (*P*<0.001).
(TIF)

Figure S6 **Viability of PBMC in response to CY2, CY14 and CY15 for 36 h.** The GI_50_ values where calculated from MTT assay Values are mean ± S.D. and represent one of the 3 representative experiments (*P*<0.001).
(TIF)
