# Supplementary figures and images for: Cytotoxic Activity and Apoptosis-Inducing Potential of Di-spiropyrrolidino and Di-spiropyrrolizidino Oxindole Andrographolide Derivatives
Source: PLoS One. 2013 Mar 5;8(3):e58055. doi: 10.1371/journal.pone.0058055 (PMC3589478; doi:10.1371/journal.pone.0058055)

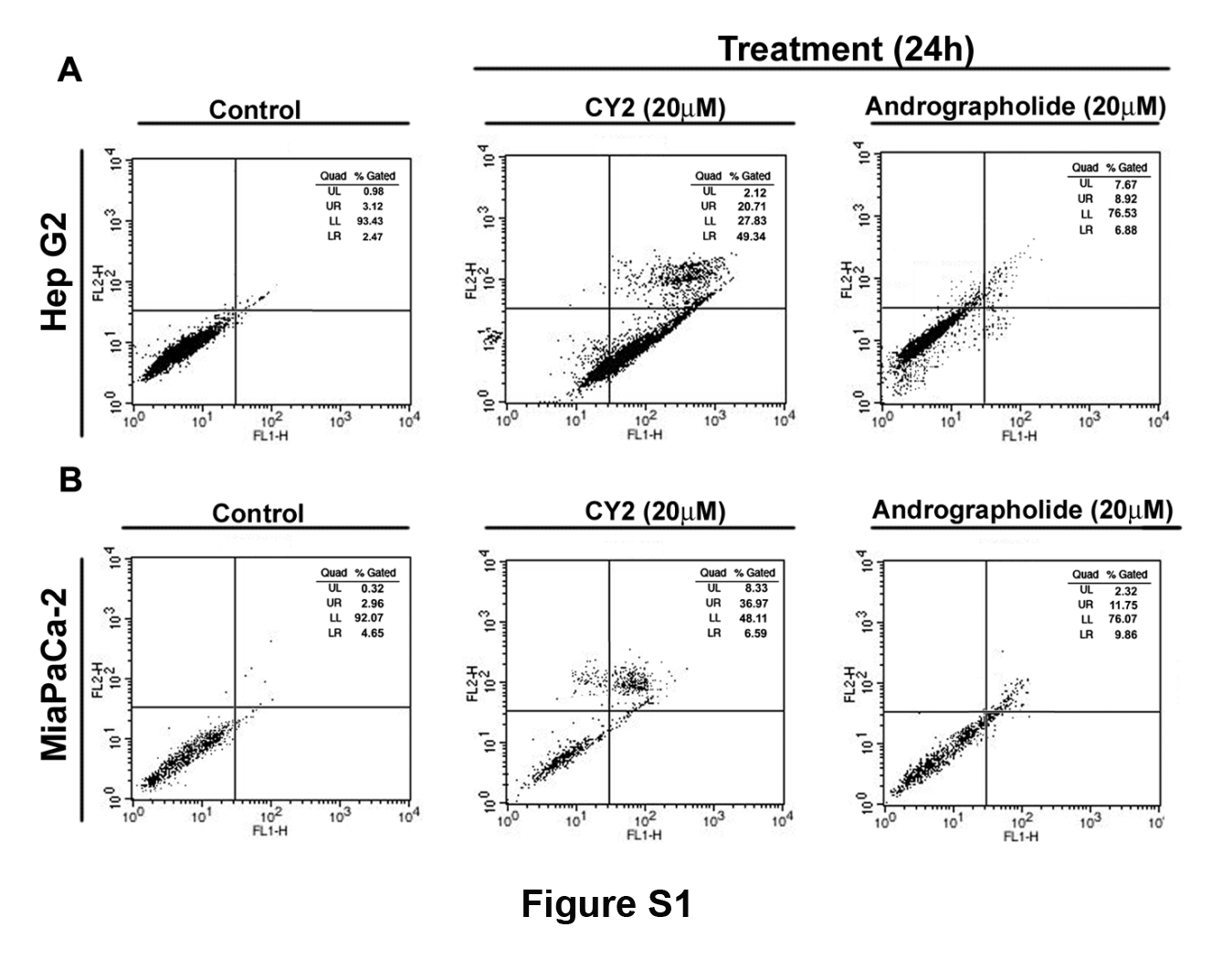

Supplement: Figure S1 — Analysis of apoptosis by flow cytometry in HepG2 and MiaPaCa-2 annexin-V/FITC. Cells were treated with 20 µM of CY2 (left panel) and andrographolide (right panel) for 24 h. Binding of annexin V to phosphatidyl serine was determined by flow cytometry. Percentages of apoptotic cells determined by the number of annexin V (+)/propidium iodide cells are shown in the scattered plot. (A) Apoptosis analysis in HepG2 cells. (B) Apoptosis analysis in MiaPaCa-2 cells. (TIF) [file pone.0058055.s001.tif]

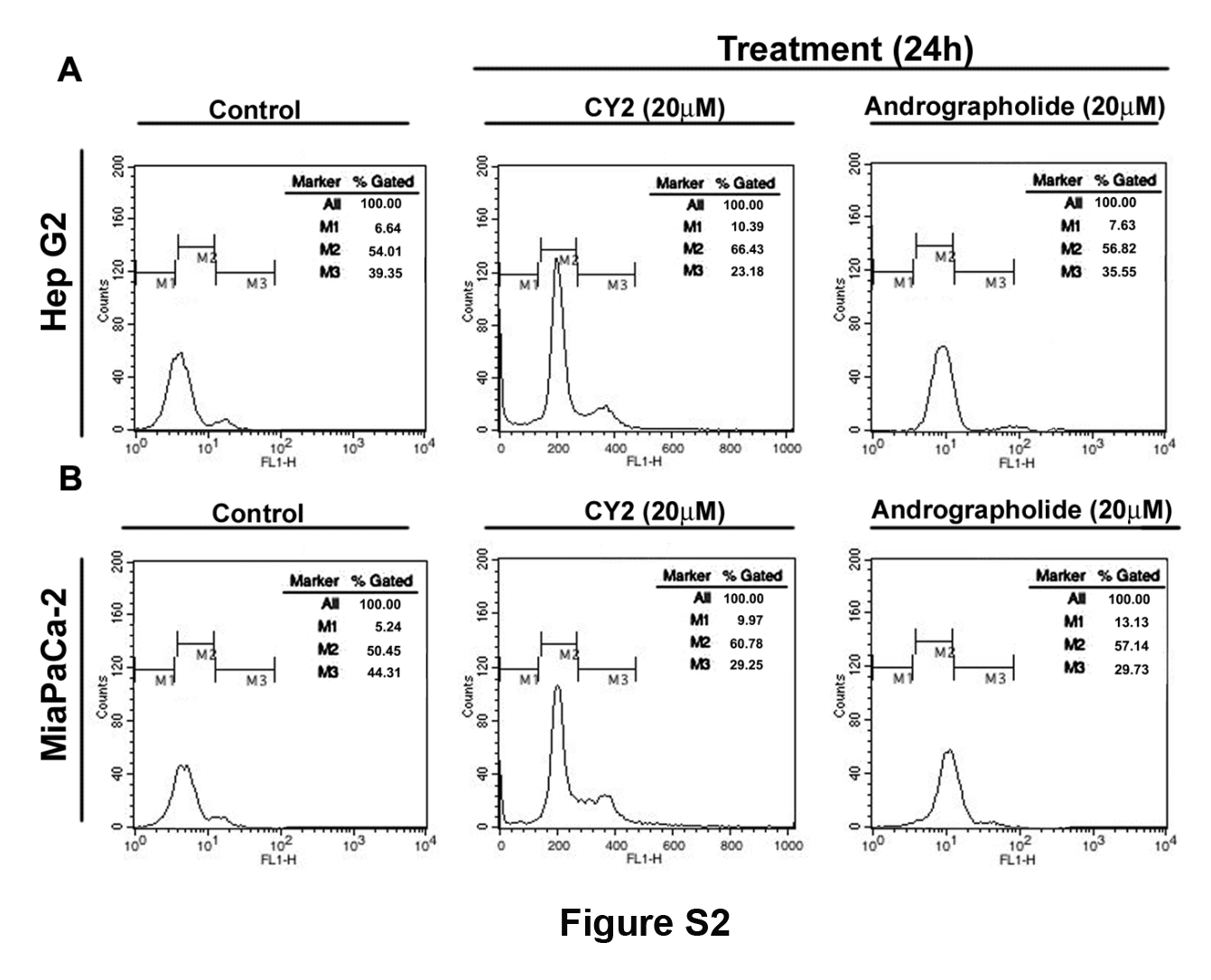

Supplement: Figure S2 — Study of cell cycle arrest by propidium iodide in HepG2 and MiaPaCa-2. Cells treated with or without 20 µM of CY2 and andrographolide for 24 h were used for cell cycle arrest study as described in materials and methods. Percentage of G0–G1 cell population increases after treatment of indicating G1/S phase cell cycle arrest. (A) Cell cycle arrest in HepG2 cells. (B) Cell cycle arrest in cells MiaPaCa-2. (TIF) [file pone.0058055.s002.tif]

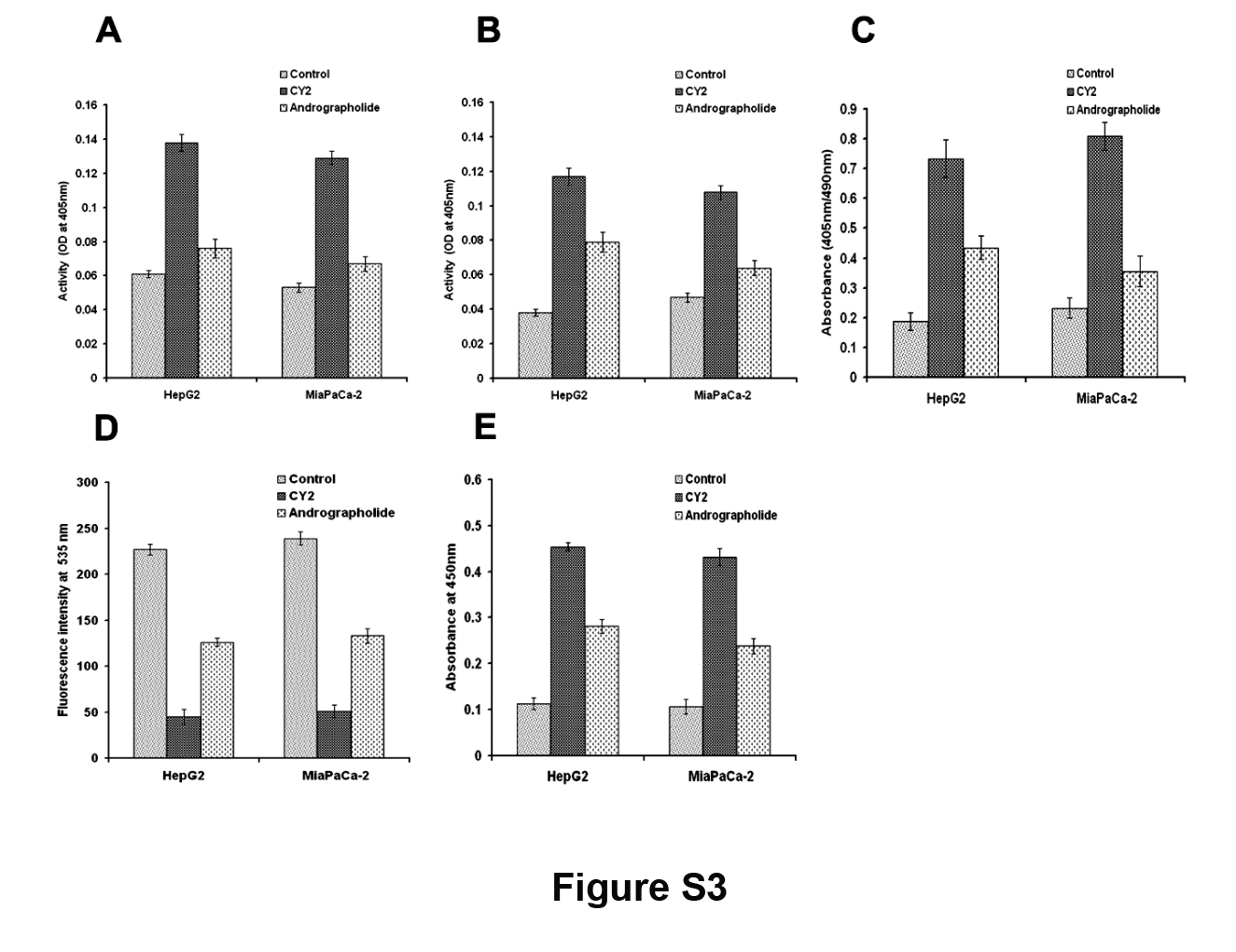

Supplement: Figure S3 — Analysis of caspase-3 and caspase-9, DNA fragmentation, mitochondrial membrane potential and cytochrome c level Cells (2×10^5^) after treated with 20 µM of CY2 for 24 h by ELISA based colorimetric assay using kits in HepG2 and MiaPaCa2. Enhancement of O.D. represents activation of (A) caspase-3, (B) caspase-9. (C) DNA fragmentation (D) Mitochondrial Membrane Potential (E) Cytochrome c level in HepG2 and MiaPaCa-2 cell lines. Values are mean ± S.D. and represent one of the 3 representative experiments (P<0.001). (TIF) [file pone.0058055.s003.tif]

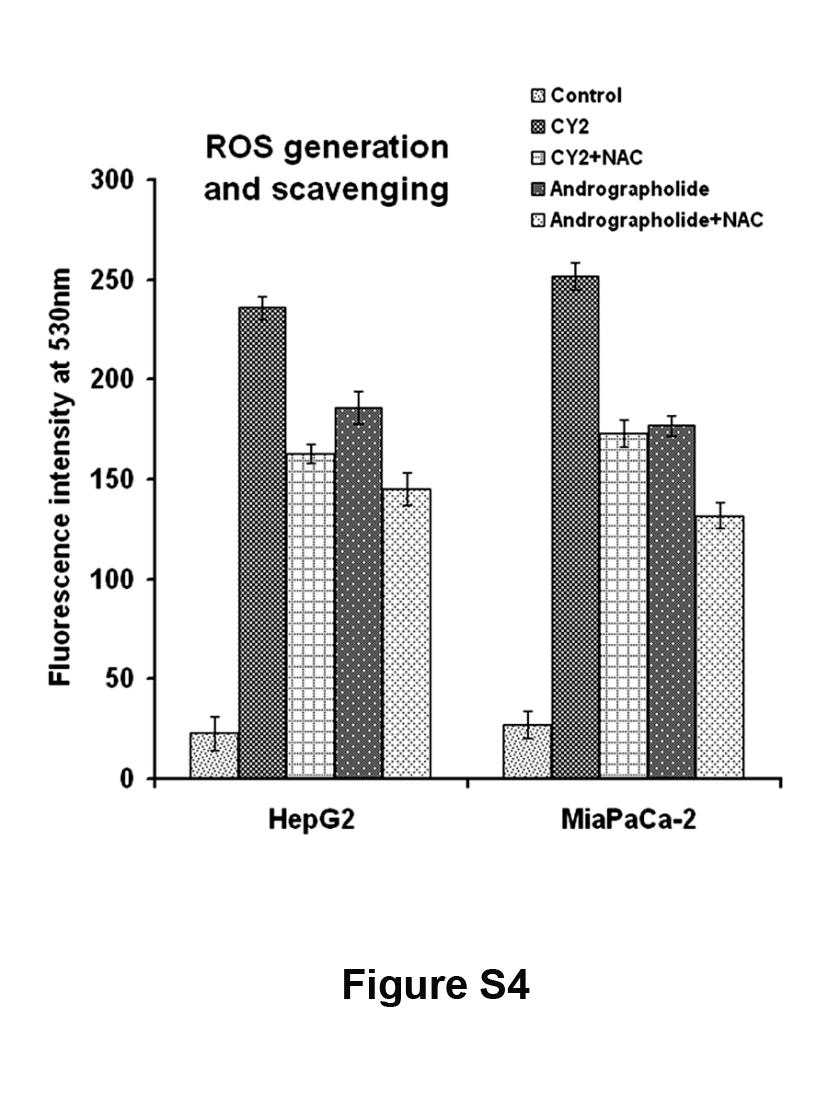

Supplement: Figure S4 — A relative ROS generation by 20 µM of CY2 and andrographolide with the scavenging action by NAC in HepG2 and MiaPaCa-2 cell lines. Flourosence intensity of 2′,7′-dichlorofluorescein (DCF) in HepG2 and MiaPaCa-2 cell lines after treatment of 20 µM of CY2 and andrographolide incubated for 24 h in treated without NAC and NAC (Values are mean ± S.D. and represent one of the 3 representative experiments (P<0.001). (TIF) [file pone.0058055.s004.tif]

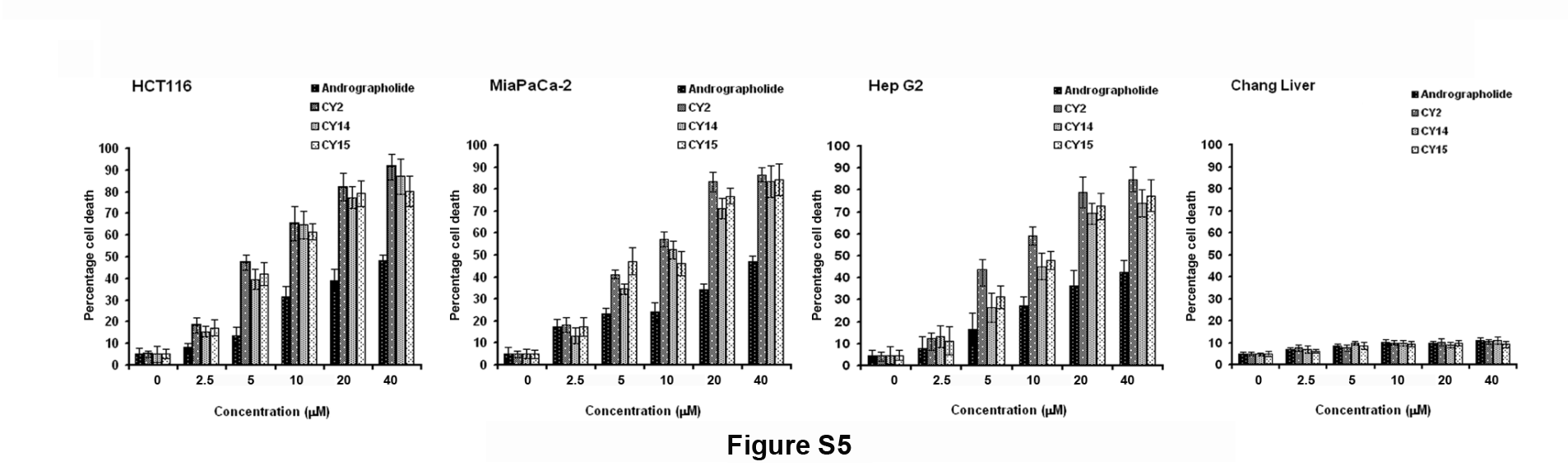

Supplement: Figure S5 — Viability of three cancer lines HCT116, MiaPaCa-2, HepG2 and a non–cancer cell line Chang liver cells was observed in response to andrographolide and its derivatives. Experimental cells (2×10^5^) were treated with andrographolide and its derivatives. Assay of dead cells by trypan blue exclusion test. Cells, untreated or treated with different concentrations of andrographolide and its derivatives of sarcosine and proline series for 36 h, were stained with trypan blue. Stained (dead) cells were counted under microscope. Number of dead cells is presented following treatment of derivatives of CY2, CY14, CY15 and andrographolide. Values are mean ± S.D. and represent one of the 3 representative experiments (P<0.001). (TIF) [file pone.0058055.s005.tif]

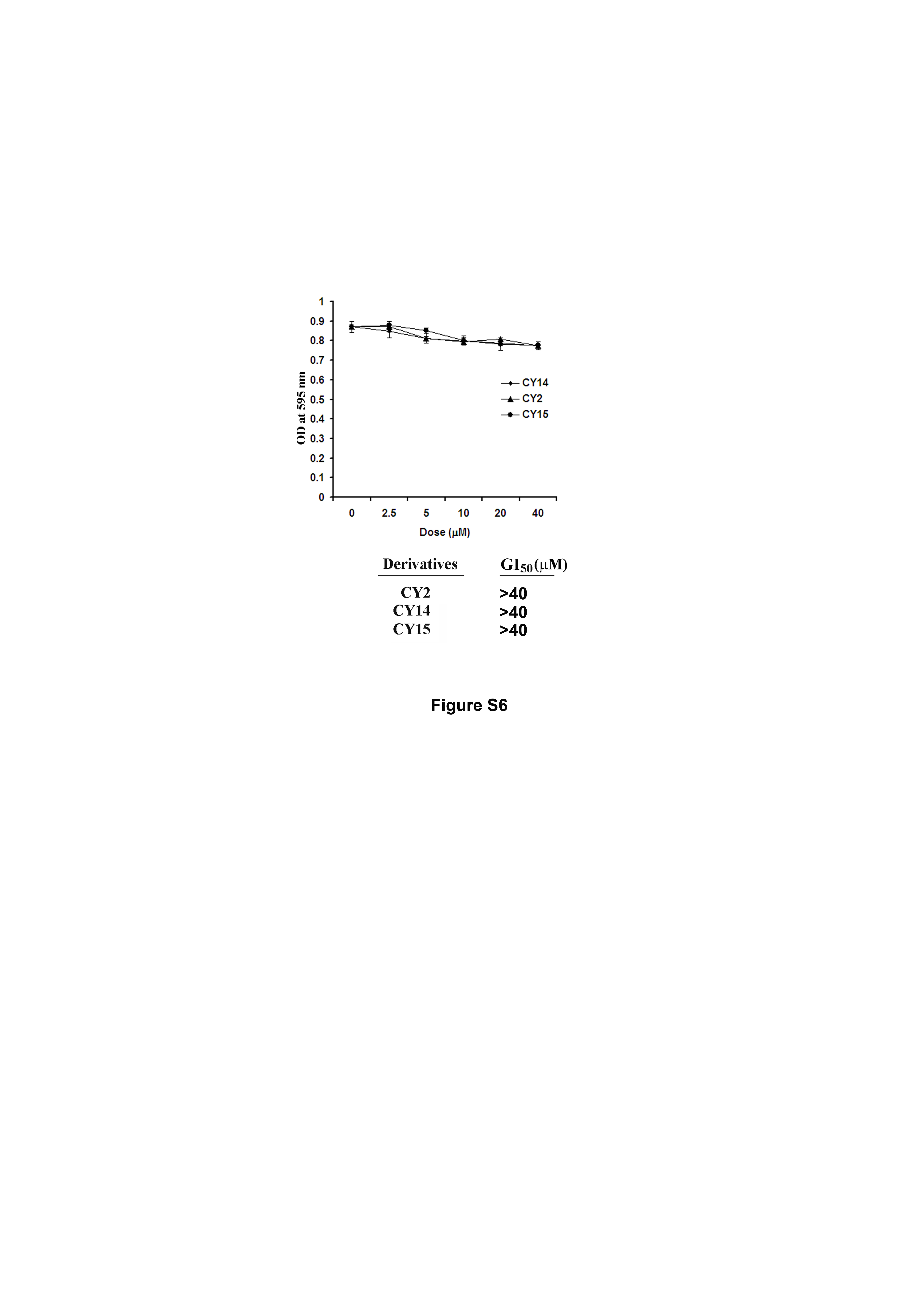

Supplement: Figure S6 — Viability of PBMC in response to CY2, CY14 and CY15 for 36 h. The GI50 values where calculated from MTT assay Values are mean ± S.D. and represent one of the 3 representative experiments (P<0.001). (TIF) [file pone.0058055.s006.tif]
